# Laparoscopic liver resection in metastatic colorectal cancer treatment: comparison with long-term results using the conventional approach

**DOI:** 10.3332/ecancer.2017.775

**Published:** 2017-10-24

**Authors:** Rafael José Maurette, Marcos García Ejarque, Matías Mihura, Mariano Bregante, Diego Bogetti, Daniel Pirchi

**Affiliations:** General Surgery Department, Hepatobiliopancreatic Surgery Section, Hospital Británico de Buenos Aires (Buenos Aires British Hospital), Buenos Aires 1280, Argentina

**Keywords:** laparoscopic liver surgery, liver surgery, colorectal cancer, liver metastases

## Abstract

**Background:**

Laparoscopic liver resections (LLRs) have been shown to be both feasible and safe. However, no randomised control studies have been performed to date comparing results with those of the open surgery approach.

**Main aim:**

To analyse LLR long-term results and compare them with a similar group of open resections in patients with colorectal carcinoma liver metastasis (CRCLM).

**Methods:**

Retrospective study on a prospective database. All patients with anatomopathological diagnosis of CRCLM resected between July 2007 and July 2015.

**Results:**

Twenty-two open resections and 18 laparoscopic resections which presented favourable lesions for laparoscopic approach were analysed. Postoperative grade III morbidity was similar in both groups (p = 0.323). Disease-free survival at 1, 3, and 8 years in the laparoscopy group (n =16) was 81%, 58%, and 58%, respectively, while in the open surgery group (n = 17) it was 64%, 37%, and 19% respectively; no differences were found (p = 0.388). Global survival in the laparoscopy group was 93%, 60%, and 40%, respectively, and 88%, 74.5%, and 58.7%, respectively, in the open surgery group; no differences were found (p = 0.893) with a 37 months average follow-up.

**Conclusion:**

LLR in patients with technically favourable CRCLM had similar morbidity to open resections and resection margins were not compromised because of laparoscopy.

## Introduction

Numerous series of descriptive reports, comparing cases and controls, meta-analyses, and innovative applications have been published [2], but no randomised prospective study (RPS) comparing an open surgery approach with a laparoscopic approach in liver resections has been published to date. Conclusions at the Second International Consensus Conference on Laparoscopic Liver Resection (ICCLLR) held in Marioka, Japan, recommend its use for specific indications but with an intermediate evidence degree [3]. Consensus was achieved for some indications such as hepatocellular carcinoma and in colorectal cancer metastasis. Nevertheless, laparoscopy is not a standard practice in all its applications, and it is still in the evaluation process considered to be an ‘innovative technology’ 2b [4].

This lack of dissemination is because of the fact that amongst other reasons it is a procedure which requires experience in hepatobiliary open surgery and in laparoscopy, implying a steep learning curve, and also because of bleeding risk, fear of not achieving adequate margins in malignant pathology, and to the lack of manual palpation of small nodules which are difficult to detect with translaparoscopic ultrasound scan.

Comparative studies published regarding both procedures for the treatment of colorectal cancer liver metastases (CRCLM) with short- and long-term (16–41 months) follow-up have not shown major differences in immediate outcomes and in survival [3].

Some of these studies defined patient’s subgroup as ‘favourable for laparoscopy’, meaning better outcomes when laparoscopy was performed. We are not sure whether these results, obtained in the United States and Europe can be replicated in South America. Given that liver resections encompass heterogeneous procedures, it is intrinsically difficult to globally evaluate them and a greater number of cases are necessary to study them. It is therefore necessary to compare outcomes in the patient group where laparoscopy is a feasible procedure.

The objective of the present study is to analyse laparoscopic liver resection (LLR) outcomes and compare them with those of a similar patient group who received open surgery resections for the treatment of CRCLM within patients classified as ‘favourable for laparoscopy.’ This comparison will address terms of safety, resection margin, and oncological results.

## Materials and methods

A retrospective study on a prospective database was performed with two series of CRCLM patients who underwent either laparoscopy or open surgery. All patients with anatomopathological diagnosis of CRCLM resected between July 2007–July 2015 were included.

The following exclusion criteria where used:

- extrahepatic metastases
- peritoneal carcinomatosis or extension to neighbouring organs
- multiple bilateral liver metastases (multiple amounting to more than five lesions)
- size > 6 cm
- two-stage hepatectomy
- re-hepatectomies
- resections with lesions close to the suprahepatic veins confluent
- trisectorectomies

As favourable for laparoscopy, we included the left lateral sectionectomies, and those patients with tumours located in segments II, III, IV, V, and VI. We also include some patients with lesions in segments I, VII, and VIII, but they must be superficial.

With a view to evaluating if both groups were comparable, demographic data listed in Table 1 were recorded.

The following variables were compared:

1. Surgery: type of hepatectomy; anatomical versus non-anatomical, major or minor hepatectomy according to Brisbane [5] classification, associated procedures on the liver, length of the hepatectomy, clamping of the pedicle and duration, transfusions and intraoperative events.
2. Short-term postoperative: liver and general complications stratified according to Dindo-Clavien’s classification [6], postoperative biliary filtration (ISGLS) [7], and hospital stay.
3. Oncological results: size and number of resected metastases, N of TNM of colon primary tumour, resection margin in pathological anatomy, classification of hepatic resection in R0 versus R1 and multimodal treatment. In the patient’s subgroup where no radiofrequency ablation was performed, variables in the medium and long term were analysed: relapse and relapse location (within or without the liver) recurrence free survival and global survival.

## Surgical technique

For open liver resections the standard technique was used as described in the bibliography.

The patient’s position for lesions located in posterior segments was a semi lateral decubitus at 45°, and for medial or anterior lesions a dorsal decubitus. The legs were ajar with intermittent pneumatic compression.

The cavity was accessed with the open technique and 12 and 5 mm trocars were placed according to the planned resection. Pneumoperitoneum was performed at 12 mmHg. The liver was moved partially or completely according to the hepatectomy type. In all cases translaparoscopic ultrasound scan was performed with ultrasound scan transducer with Sonosite® or Aloka® flexible tip. When the anatomic hepatectomy was planned, a routine transcystic intraoperative cholangiography was performed in order to get to know the biliary anatomy.

Before and during liver transection, liquid infusion was restricted in order to keep CVP below 5 mmHg and using inotropic agents when it was necessary to keep MAP below 70 mmHg. Ventilator values were adjusted to reduce current volume; respiratory frequency was increased in order to keep an adequate pPO2. This anesthetic procedure and approach, called ‘caudal approach’ has been described by Soubrane *et al* [8].

In right or left hepatectomies intra glissonian or extra glissonian approaches were used. For parenchymal transection, an Ultrasicion® ultrasonic one vessel sealer and bipolar electro sealer scalpel, Kellyclasia procedures and titanium or Hem-o-lock clips according to vessel caliber were used. Homeostasis was completed, if necessary with ‘x’ stitches in V-Lock® suture or Prolene® sutures. If there was bleeding at the subhepatic vein, pneumoperitoneum pressure was temporarily increased to 20 mmHg until bleeding was controlled. In order to finish transection, haemostatic plates or plastic glue were used in some cases. Silicone drainage was provided to the resection bed.

The pieces were extracted with a bag through umbilical midline incision or through Pfannestiel incision.

### Statistics analysis

Continuous data are expressed as median or standard deviation (SD) with the corresponding range between parentheses. The Mann–Whitney U test was used for comparing continuous data, while the X^2^ test was used for categorical data. The Kaplan-Meier test was used to analyse survival curves and the log-rank test to look for statistically significant differences between them. The significant value taken was p<0.05. All statistic analyses were made with IBM SPSS Statistic 20.0® software.

## Results

During the study period 128 hepatectomies were performed, 49 of which were LLR, giving an applicability rate of 38.2%. In the same period 67 liver resections because of CRCLM were performed, 41 by open surgery and 26 by laparoscopy. Once the previously mentioned exclusion criteria were applied, the population groups were made up of 22 open surgery resections and 23 laparoscopies. Out of the latter five turned into open surgery: one because of bleeding, two because more nodes than had been foreseen were found, and the last two because the location was difficult to access. The five changeovers to open surgery took place in the first half of the series. No changeovers were because of the need to enlarge margins.

Ten patients, five from laparoscopy and five from open surgery underwent liver resection at the same time as colon surgery.

Therefore, the patient population for this study amounted to 22 resections, performed simultaneously with laparoscopic colectomy, using the hand assisted technique with Alexis® retractor placed at the mini laparotomy where the resected colon was extracted.

No statistically significant differences were found in the demographic data for both groups, except for the node size in imaging studies; larger size nodes were found in the open surgery group (22.5 mm versus 29 mm) as can be seen in Table 1.

Table 2 shows surgical variables for both groups. The following results are highlighted: in two laparoscopic resections a liver radiofrequency ablation (RFA) procedure was simultaneously performed and five in the open surgery group, but this difference was not statistically significant (p = 0.336). Length of the surgical procedure was shorter for laparoscopies compared with open surgeries (245 min versus 300). This was the only statistically significant difference of all intraoperative variables between both groups (p = 0.0310).

### Short-term results

No differences were found in morbidity indexes between both groups.

Complications according to Dindo-Clavien’s classification are presented in Table 3. Morbidity grade 4 was a duodenal perforation in the laparoscopy group and an enteric fistula in the open surgery group.

No significant differences were found in the number of major complications, grade 3 or 4 according to Dindo-Clavien’s classification in liver morbidity and in biliary filtration. No mortality took place in the series.

### Oncological results

Average follow-up is 37.7 months (8 to 98 months). Only one patient was lost for follow-up in the open surgery group.

As can be seen in Table 4 no differences were found in oncological risk factors such as poor differentiation, nodule size and number, distance from the resection margin, resection R0 or R1, and N colon primary tumour. More patients in the open surgery group received neoadjuvant chemotherapy (p = 0.028). This was because of the oncological protocol in our hospital whereby patients with synchronic presentations and undergoing simultaneous procedures for the primary tumour and metastases do not get neoadjuvant chemotherapy but adjuvant chemotherapy.

**Figure 1.**
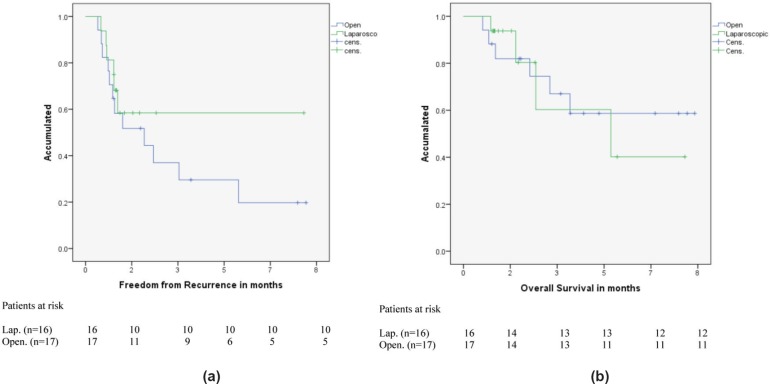
Free from disease survival 1, 3, and 8 years. Resections without RFA, Laparoscopy group N = 16, Open surgery N = 18.

Excluding patients who received RFA, free from disease survival in 1, 3, and 8 years in the laparoscopy group (n = 16) was 81% (NS 0.098), 58% (NS 0.135), and 58% (NS 0.135); and in the open surgery group (n = 17) 64% (NS 0.116), 37% (NS 0.125), and 19% (NS 0.113); no statistically significant differences were found. (p = 0.388). Global survival was 1, 3, and 8 years in the laparoscopy group (n = 16) was 93% (NS 0.061), 60% (NS 0.201), and 40% (NS 212); and in the open surgery group (n = 17) it was 88% (NS 0.078), 74.5% (NS 0.112), and 58.7% (NS 0.133); no statistically significant differences were found (p = 0.893) (Figure 1).

Exclusive liver relapse was 75% (3/4) in the laparoscopy group and 41% (5/12) in the open surgery group.

## Discussion

The number of LLRs performed around the world has increased of late, mainly in the United States, Europe, and Eastern countries. This greater diffusion of laparoscopy in liver surgery is in part due to the important progress achieved in the technology and in anesthetic procedures. The concept of ‘caudal approach’ and ‘T approach,’ control of insufflation pressures of the pneumoperitoneum, high definition magnification of the surgical field, together with new skills in intracorporeal stitches and knots, added to the adequate handling of new anesthetic variables have made it possible to handle intraoperative bleeding, one of the great fears with this technique [2, 3, 8, 11]. Nevertheless, in malignant pathology there is also the worry of not being able to adequately resect oncological margins. One of the main reasons is lack of manual palpation and the steep learning curve in handling translaparoscopic sound scans [12^].^ Yet another negative consequence is the deep margin of non-anatomical resections which is the same as in open surgery but increased by limitations of straight laparoscopic instruments.

After Nguyen *et al’s* publication in 2009 [13], numerous studies have been published about a series of cases of LLR in the treatment of CRCLM and comparative studies between laparoscopies and open surgery procedures. At the second International Consensus Conference on Laparoscopic Liver Resection (ICCLLR) [3], the conclusion was that LLRs had a similar mortality rate as open surgery procedures, that morbidity has decreased in certain areas, that margins obtained are not inferior to those in open surgery and that there are no differences in free from recurrence survival and global survival when compared with open surgery procedures. But when open series of laparoscopies are compared for the treatment of tumours, there is a selection bias at the expense of open surgery which raises the need for comparisons with strict exclusion criteria in order to draw valid conclusions. It is also important to compare both groups according to the main oncological risk factors for CRCLM, such as size >5, number, bi-laterality, synchronicity, and N of the primary tumour[14]. In our analysis there were no differences in these variables in both groups, with the exception of node size in sound scans, but this difference was not reflected when nodes were examined in anatomical pathology.

Our study, being retrospective, was probably biased in terms of selection. That is why a less ambitious objective was defined: to exclusively analyse cases which were ‘favourable for laparoscopy’ operated on both with laparoscopy and with open surgery. Therefore, strict exclusion criteria were set forth in terms of size, number, and location of nodes in the so-called laparoscopic or peripheral segments (S2, S3, S4b, S5, and S6). Nevertheless, Ban *et al’s* publication should be underscored: a difficulty scoring system was proposed whereby cases were stratified according to their degree of difficulty for laparoscopic technique [15]. This study shows that a selection bias could still be present as some surgeons might consider laparoscopy not suitable for hepatectomies while others might think it is.

As we adopted very strict inclusion criteria, only patients with CRCLM ‘favourable for laparoscopy,’ the sample size was small. But if this study is compared with most of the published international series, several of these studies have the same number of cases, 20 to 25, but not all of them adopted such strict selection criteria. Table 5 [16–25].

In our study we did not find differences in global morbidity, or of the liver itself, or in the number of fistulas as defined by ISGLS. At the beginning of the series there was a case of intraoperative bleeding in the laparoscopy group which required changeover to open surgery. At present there are no differences in intraoperative events or in the number of transfusions in both groups. Hospital stay showed no statistically significant differences, but it should be noted that prompt recovery inherent to laparoscopic procedures did not delay the beginning of adjuvant chemotherapy.

The most important outcome of this study is to confirm that margins achieved were equal or better than those in open surgery. Nevertheless, we should stress the fact that this applied to a group which was ‘favourable for laparoscopy,’ therefore, we cannot extrapolate this conclusion to all liver resections. One of the patients in the laparoscopy group R1, whose freezing biopsy during surgery was informed as negative margin by macroscopy, eventually turned out to be deep margin in the deferred examination. This situation of negative margins in the immediate freezing and positive in the deferred examination can also take place in open surgery. No statistically significant difference was found in free from disease survival and in global survival during an eight year follow-up. This study is not aimed at showing oncological advantages in laparoscopic resection as there could be biases which have not yet been analysed, but it does show that in terms of safety, resection margin and oncological outcomes, laparoscopy is similar to open surgery.

## Conclusions

Our study shows that LLRs in patients with technically favourable CRCLM, apart from inherent advantages of the mini-invasive approach in recovery and parietal morbidity, had similar morbidity rates as open surgeries, resection margins were not compromised by laparoscopy, and despite possible selection bias, global and free from recurrence survival was similar to the laparoscopy group in the long term. Even though the objective of our study was not to establish the advantages of laparoscopy, these outcomes allow us to recommend laparoscopy for this patient group without fears associated to potential negative effects in surgical and oncological safety in this procedure.

## Figures and Tables

**Table 1.** Demographic data in the laparoscopy group and in the open surgery group.

Variable	Laparoscopy Group (n=18)	Open surgery group (n=22).	p =
Sex F/M	7/11	7/15	0.641
Age, median (range)	66 (33–83)	58.7 (33–78)	0.059
B.M.I., median (range)	27.2 (19.6–33.2)	26.6 (18.7–34.5)	0.690
ASA (I/II/II)I	0/9/9	3/12/7	0.191
CEA, median (range)	3.15 (1.6–375)	10 (1–335)	0.236
Node size in mm, median (range)	22.5 (7–60)	29 (12–60)	0.0497
Bilateral	7/18	10/22	0.676
Node number, median (range)	2 (1–4)	2.5 (1–5)	0.168
Synchronic/metachronous	9/18	8/22	0.385
Segments NOT laparoscopic*	12/18	12/22	0.436

* S7, S8, S4A

**Table 2.** Operative variables.

Variable	Laparoscopy group(N=18)	Open surgery group (N=22)	p =
Hepatect. Anat/No anat/Anat+No anat	8/8/2	5/12/5	0.301
Major Hepatectomy	4	7	0.499
Associated RFA	2	5	0.336
Min. surgery time, median (range)*	245 (75–510)	300 (165–590)	0.0310
Pringle’s manoeuvre	10/18	9/22	0.429
Min. Duration Pringle’s manoeuvre, median (range)	36 (15–50)	27.5 (13–50)	0.205
UGR Transfusion (n° of patients)	3/18	8/22	0.165
UGR Transfusion (n° of units), median (range)	2 (2–6)	2 (1–7)	0.599
Intraoperative Events	0	1	0.265
Hospital Stay	5 (4–49)	7 (4–73)	0.0852

* Only hepatectomy.

**Table 3.** Global and specific morbidity for both groups.

	Complication	Laparoscopy group N = 18	Open surgery group N = 22	p =
**Grade 1–2**	Pneumonia	1	2	
	Infection of the wound	1	2	
	Biliary fistula A	2	1	
**Grade 3A and 3B**	Abdominal abscess*	0	3	
	Fistula B	2	1	
	Fistula C	1	2	
**Grade 4**	Hollow Viscera Perforation	1	1	
**Complications > 3**		2	6	0.234
**Liver Complications**		5	4	0.470
**Fistula**^£^	A	2	1	
	B	2	1	
	C	1	2	0.701

* Percutaneous drainage was required.

£ International Study Group of Liver Surgery (ISGLS)

**Table 4.** Oncological variables.

Variable	Laparoscopy Group (N = 18)	Open surgery group (N = 22)	p =
Histology Adenocarcinoma Mucus-secreting adenocarcinoma Complete necrosis	15 2 1	17 5 0	0.362
N° of nodes (range)	2 (1–7)	2 (0–7)	0.635
Size of the biggest node in mm, median (range)	26 (5–100)	26.5 (11–64)	0.692
Histological Grade G3^£^	3/18	1/22	0.310°
	4.5 (0–40)	5 (1–23)	0.726
Resection R0/R1*	1/16	0/17	0.295
N of primary tumour N0/N1/N2	6/8/4	7/13/2	0.461
Neoadjuvant chemotherapy	6/18	15/22	0.028
Adjuvant chemotherapy	16/18	14/14	0.492°

£ G3: poorly differentiated.

* Patients with RFA associated to them were not included.

° Fischer

**Table 5.** Publication of a series of comparative cases between laparoscopy and open Surgery for liver resection in CRCLM.

Author	Year	N° pat.	Follow-up months	RFS months	Three years RFS (%)	Five years RFS (%)	Three years GS (%)	Five years GS (%)	p =
		**RHL / RHA**	**RHL / RHA**	**RHL / RHA**	**RHL / RHA**	**RHL / RHA**	**RHL / RHA**	**RHL / RHA**	
**Castaing**	2009	60/60	30/33	47/40	47/40	35/27	82/70	64/56	NS
**Nguyen**	2011	24/25	27/29	NA	63/46	NA	75/79	NA	NS
**Hu**	2012	13/13	NA	NA	NA	NA	55/54	27/31	NS
**Topal**	2012	20/20	NA	NA	NA	43/23	NA	48/46	NS
**Cheung**	2012	20/40	NA	9.8/10.9	42/18	42/18	54/65	54/22	NS
**Cannon**	2012	35/140	NA	NA	37/39	15/22	63/60	36/42	NS
**Qiu**	2013	30/30	NA	NA	NA	NA	NA	NA	
**Guerron**	2013	40/40	16/16	23/23	NA	NA	89/81	NA	NS
**Iwahashi**	2013	21/21	NA	NA	14/33	14/25	84/89^£^	42/51	NS
**Montalti**	2014	57/57	41/54	NA	39/42	29/38	75/75	60/65	NS
**HB**	2015	24/24	24/35	14/24	29/25	29/18	55/49	40/39	NS

* Follow-up in months.

£ Follow-up in two years.

## References

[1] Gagner M, Rogula T, Selzer D (2004). Laparoscopic liver resection: benefits and controversies. Surg Clin North Am.

[2] Nomi TT (2015). Totally laparoscopic right hepatectomy combined with resection of the inferior vena cava by anterior approach. Ann Surg Oncol.

[3] Wakabayashi G (2015). Recommendations for laparoscopic liver resection: a report from the second international consensus conference held in Morioka. Ann Surg.

[4] McCulloch PP (2009). No surgical innovation without evaluation: the IDEAL recommendations. Lancet.

[5] Strasberg SM (2005). Nomenclature of hepatic anatomy and resections: a review of the Brisbane 2000 system. J Hepatobiliary-Pancreat Surg.

[6] Dindo D, Demartines N, Clavien PA (2004). Classification of surgical complications: a new proposal with evaluation in a cohort of 6336 patients and results of a survey. Ann Surg.

[7] Rahbari NN (2011). Post-hepatectomy haemorrhage: a definition and grading by the International Study Group of Liver Surgery (ISGLS). HPB (Oxford, England).

[8] Soubrane O (2014). A conceptual technique for laparoscopic right hepatectomy based on facts and oncologic principles: the caudal approach. Ann Surgery.

[9] Juan Pekolj RSC (2008). Resecciones hepáticas por vía laparoscópica. Experiencia inicial. Rev Argent Cirug.

[10] Maurette R (2015). Abordaje laparoscópico en el manejo de lesiones sólidas de hígado. Experiencia inicial.

[11] Wakabayashi G (2014). Laparoscopic hepatectomy is theoretically better than open hepatectomy: preparing for the 2nd International Consensus Conference on laparoscopic liver resection. J Hepatobiliary-Pancreat Sci.

[12] Vigano L (2009). The learning curve in laparoscopic liver resection: improved feasibility and reproducibility. Ann Surg.

[13] Nguyen KT, Gamblin TC, Geller DA (2009). World review of laparoscopic liver resection-2,804 patients. Ann Surg.

[14] Spelt L (2012). Prognostic models for outcome following liver resection for colorectal cancer metastases: A systematic review. Eur J Surg Oncol.

[15] Ban DD (2014). A novel difficulty scoring system for laparoscopic liver resection. J Hepatobiliary-Pancreat Sci.

[16] Castaing DD (2009). Oncologic results of laparoscopic versus open hepatectomy for colorectal liver metastases in two specialized centers. Ann Surg.

[17] Nguyen KT (2011). Comparative benefits of laparoscopic vs open hepatic resection: a critical appraisal. Arch Surg.

[18] Hu MG (2012). Outcomes of open versus laparoscopic procedure for synchronous radical resection of liver metastatic colorectal cancer: a comparative study. Surg Laparosc Endosc and Percutan Tech.

[19] Topal B (2008). Laparoscopic versus open liver resection of hepatic neoplasms: comparative analysis of short-term results. Surg Endosc.

[20] Cheung TT (2013). Outcome of laparoscopic versus open hepatectomy for colorectal liver metastases. ANZ J Surg.

[21] Cannon RM (2012). Laparoscopic versus open resection of hepatic colorectal metastases. Surgery.

[22] Qiu JJ (2013). Laparoscopic hepatectomy for hepatic colorectal metastases – a retrospective comparative cohort analysis and literature review. PloS One.

[23] Guerron ADAD (2013). Laparoscopic versus open resection of colorectal liver metastasis. Surg Endosc.

[24] Iwahashi SS (2014). Laparoscopic hepatic resection for metastatic liver tumor of colorectal cancer: comparative analysis of short- and long-term results. Surg Endosc.

[25] Montalti RR (2014). Laparoscopic liver resection compared to open approach in patients with colorectal liver metastases improves further resectability: oncological outcomes of a case-control matched-pairs analysis. Eur J Surg Oncol.

